# Concentration and Specialty Pair Patterns of Interdepartmental Consultations in Hospitalized Patients Using Real-World Data: Retrospective Cohort Study

**DOI:** 10.2196/81670

**Published:** 2026-07-14

**Authors:** Li Zhang, Shuo Liu, Ling Lan, Haiqiong Zhang, Xin Luo, Dongxu Zhao, Xiaochen Xu, Minghui Li, Ling Qiu

**Affiliations:** 1 Office of Planning and Development, Peking Union Medical College Hospital, Chinese Academy of Medical Science and Peking Union Medical College, Dongdan, Shuaifuyuan No.1, Beijing, China; 2 Department of Ultrasound, Peking Union Medical College Hospital, Chinese Academy of Medical Science and Peking Union Medical College, Beijing, China; 3 Department of Anesthesiology, Peking Union Medical College Hospital, Chinese Academy of Medical Science and Peking Union Medical College, Beijing, China; 4 Department of Nuclear Medicine, Peking Union Medical College Hospital, Chinese Academy of Medical Science and Peking Union Medical College, Beijing, China; 5 Department of Urology, Peking Union Medical College Hospital, Chinese Academy of Medical Science and Peking Union Medical College, Beijing, China; 6 Administration Office, Cangzhou Central Hospital, Cangzhou, China

**Keywords:** interdepartmental consultation, multidisciplinary collaboration, real-world data, pareto distribution, disease-driven

## Abstract

**Background:**

Interdepartmental consultations are essential for managing complex inpatient care but are often inefficient. Hospital-wide, data-driven analyses are needed to guide process improvements; yet, most existing studies have focused on single departments or specific diseases, leaving a gap in understanding hospital-level collaboration networks. Understanding these patterns is crucial for optimizing clinical workflows, reducing delays, and improving patient outcomes in large tertiary hospitals.

**Objective:**

This study aimed to analyze the distribution and network characteristics of interdepartmental consultations across a large tertiary hospital, focusing on high-frequency collaboration pairs and their disease associations.

**Methods:**

This retrospective cohort study included all interdepartmental consultations for inpatients and emergency patients at Peking Union Medical College Hospital (Beijing, China) from January 1 to December 31, 2024. Secondary data were extracted from the Hospital Information System. In total, 102,858 valid consultations involving 42 clinical departments were analyzed. Outcome measures included consultation requests and receptions per department, per capita request intensity, and pairwise collaboration volume. High-frequency collaboration pairs were defined as those with an annual consultation volume of 300 or more. Descriptive statistics (medians and IQRs) and proportions with 95% CIs were used.

**Results:**

Consultation activity exhibited marked concentration. The Emergency Department issued the most consultation requests (19,698/102,858, 19.15%), far exceeding the median departmental request volume of 1899.5 (IQR 1359.5‐3759.25). Meanwhile, the Internal Medicine Consultation Service received the highest number of consultations (10,428/102,858, 10.14%), substantially above the median reception volume of 1886.0 (IQR 521.25‐4056.75) across departments. The per capita request intensity varied widely, with Critical Care Medicine highest value (21.64) vs a hospital-wide average of 0.32. Collaboration demonstrated a strong Pareto distribution: the top 5.37% (65/1210) of department pairs (65 pairs) accounted for 42.02% (43,221/102,858) of all consultations. These high-volume pairs were predominantly disease-specific. Examples include: Endocrinology-Ophthalmology primarily for diabetic and thyroid eye disease (1287/102,858, 1.25%); General Surgery-Otolaryngology mainly for preoperative thyroid airway assessment (1225/102,858, 1.19%); General Surgery-Clinical Nutrition for perioperative support (1032/102,858, 1.00%); Endocrinology-Clinical Nutrition for metabolic disease management (703/102,858, 0.68%); Orthopedics-Rehabilitation Medicine for postoperative rehabilitation (686/102,858, 0.67%); Oncology Medical Center-Clinical Nutrition for nutrition support in patients with cancer (681/102,858, 0.66%). Recurring clinical scenarios generated these stable, predictable consultation pathways.

**Conclusions:**

This study provides a novel, hospital-wide, network-based mapping of interdepartmental consultations using real-world data. Unlike prior work limited to single departments or diseases, it reveals that collaboration is concentrated, Pareto-like, and disease-driven. The identification of stable, disease-specific consultation pairs offers a data-driven framework for understanding multidisciplinary collaboration. These findings offer a data-driven framework for understanding multidisciplinary collaboration as a networked system. In practice, administrators and clinicians can use this evidence to prioritize resources, design standardized multidisciplinary team pathways, and implement spatial or digital interventions to reduce delays and improve patient flow and outcomes.

## Introduction

Interdepartmental consultation refers to the activity in which health care professionals from outside the originating department are invited to assist in providing diagnostic or therapeutic opinions, or to offer diagnostic or treatment services. It serves as a core mechanism for managing complex conditions, ensuring patient safety, and improving the quality of care [1-5]. In large general hospitals, patients often have complex conditions and multiple comorbidities, making effective interdepartmental consultations crucial for maintaining the continuity and integrity of diagnosis and treatment [6-11].

The clinical importance of interdepartmental consultations has been widely recognized in previous studies. For instance, multidisciplinary consultations have been shown to improve outcomes in specific disease contexts, such as sudden cardiac death, postintensive care management, rectal cancer, and diabetic foot care [2,4,8,10]. Furthermore, studies have demonstrated that consultation processes can influence length of stay, hospitalization costs, and emergency department crowding [12-16]. Several investigators have also analyzed consultation patterns within single departments or for particular diseases, including cardiology, pulmonology, endocrinology, and dermatology, offering detailed accounts of collaboration patterns, common reasons for referral, and barriers to efficiency within those specialty contexts [10,17-20].

Despite the confirmed clinical value of consultations, in practice, many hospitals face operational challenges such as delayed consultation responses and low coordination efficiency [19,21-23]. These issues can impact the timeliness of clinical decision-making, prolong hospital stays, increase the burden on medical resources, and ultimately affect health care service efficiency and patient experience [7,12,15,19,24-29]. However, most studies focus on isolated departments or specific conditions, lacking a hospital-wide, macro-level analysis of interdepartmental consultation networks using real-world data [4,6,8,20]. This gap leaves hospital administrators and clinicians unable to grasp overall collaboration patterns, identify bottleneck departments, or design evidence-based interventions to improve consultation efficiency and patient flow [7,26,30].

Therefore, systematically analyzing the practical operational characteristics of intrahospital consultations holds significant importance for optimizing clinical pathways and improving hospital operational efficiency.

Specifically, the analysis of hospital-wide consultation data can support several practical aspects of care and hospital management. It helps identify common collaboration patterns and related diseases, enabling research into the value of multidisciplinary consultations for complex conditions, such as by examining interactions between gastroenterology, nutrition, and general surgery for gastrointestinal cases [6,10,18,31-33]. Operationally, it highlights high-demand or bottleneck departments, informing workforce planning and resource allocation. Clinicians, administrators, and designers can use these data to explore ways to shorten consultation response times and enhance consultation processes, potentially improving patient flow and outcomes [26,30,34-37]. Additionally, amid China’s ongoing promotion of smart hospital development, this information can guide the design of more efficient layouts—for example, by positioning frequently collaborating departments closer together and optimizing the flow pathways for both staff and patients, which may reduce unnecessary movement and streamline workflows [38-42].

This study, based on over 100,000 valid consultation records from Peking Union Medical College Hospital (PUMCH) in 2024, aims to systematically analyze the distribution characteristics and collaboration networks of interdepartmental consultations for inpatients and emergency patients in a large general hospital. The innovation of this study lies in being the first to integrate multidimensional consultation data at the hospital-wide level, constructing a departmental collaboration network map and systematically quantifying the distribution patterns of collaboration intensity and disease-related characteristics. This research may provide a foundation for exploring the value of multidisciplinary consultation in managing complex diseases and for conducting in-depth studies, such as optimizing consultation processes and spatial layouts to improve staff and patient flow.

## Methods

### Inclusion and Exclusion

All consultations submitted and executed through the Hospital Information System (HIS) for inpatients and emergency department patients at PUMCH between January 1, 2024, and December 31, 2024, were included, totaling 145,136 consultations. This comprised 125,438 consultations for inpatients, 1439 consultations for emergency observation patients, and 18,259 consultations for emergency walk-in patients.

Outpatient consultations were excluded. Emergency walk-in patients were included because consultations requested for them also require consulting physicians from the respective departments to physically visit the patient’s bedside. Consultations where the receiving department was a diagnostic or therapeutic platform department (eg, Department of Radiology or Ultrasound) were excluded (n=23,839). The primary reason for exclusion was that physicians in these departments do not need to visit the patient’s bedside; they can provide consultation opinions directly after reviewing imaging studies in the HIS. Consultations requested from or received by comprehensive disease units (eg, International Medical Services and Clinical Research Wards) were also excluded (n=18,439). The main reason was that these units do not differentiate based on specific disease types or organ systems, making them unsuitable for disease-specific consultation analysis. Ultimately, consultations involving 42 clinical departments were included, resulting in 102,858 valid interdepartmental consultations.

### Sampling Procedures

All eligible consultation records from January 1, 2024, to December 31, 2024, were included (full sample). Data were collected at PUMCH (Beijing, China). All data were extracted from the HIS as secondary data. The HIS requires complete entry of all mandatory fields (eg, requesting department, receiving department, patient identifier, and consultation time) for each consultation to be formally executed. Consequently, all 102,858 included consultations had complete data on all variables analyzed in this study.

### Measures and Covariates

**Figure 1.**
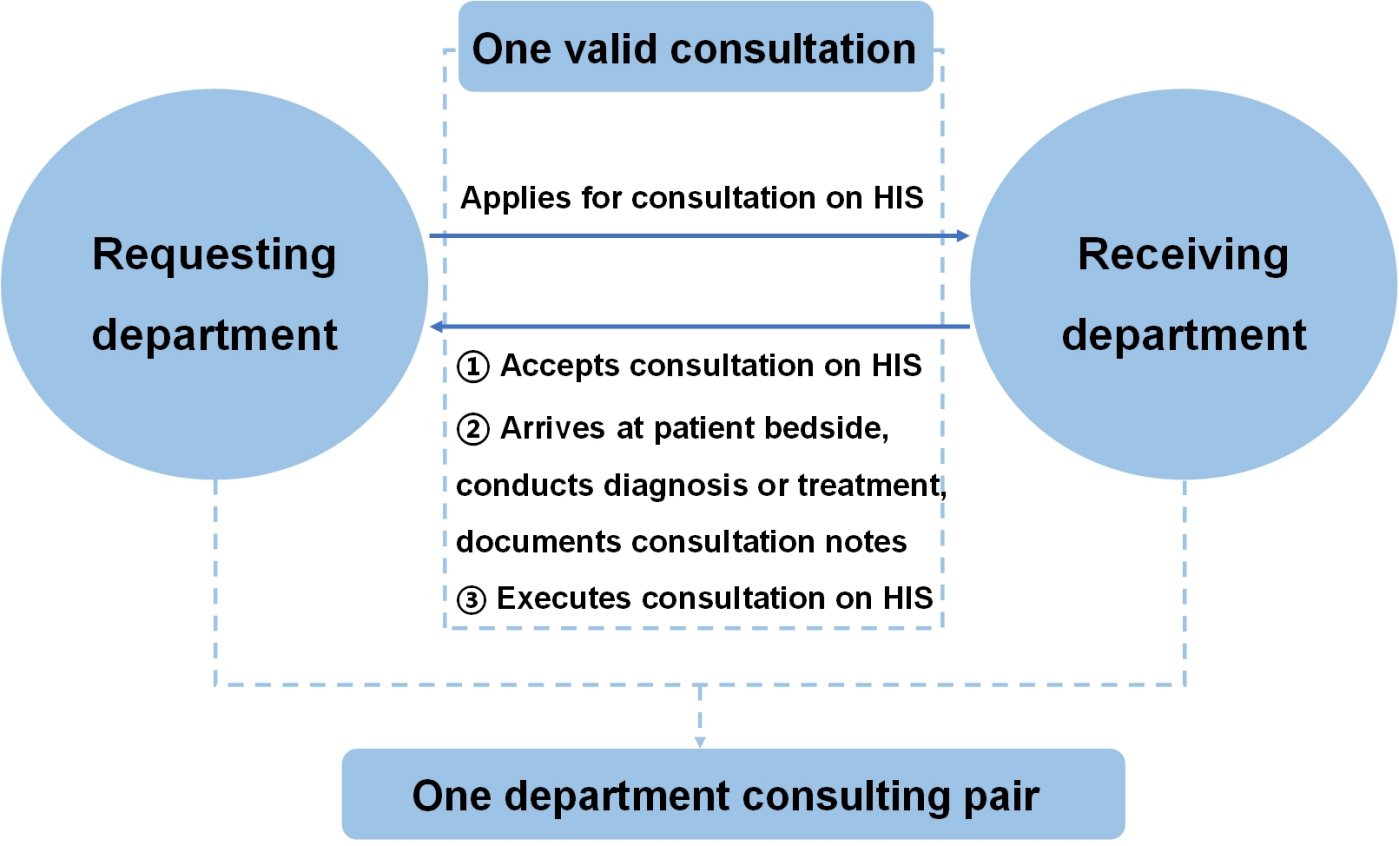
Definition of a valid consultation and a department consultation pair. In this retrospective analysis at Peking Union Medical College Hospital (Beijing, 2024), a valid consultation required Department A to submit a request via Hospital Information System (HIS) and a physician from Department B to physically visit the ward to provide an opinion. The ordered pair (A, B) defines a consultation pair.

A consultation was operationally defined as a process initiated when Department A submitted a consultation request via the HIS. Upon receipt of this request, Department B assigned a physician who physically traveled to the inpatient ward of Department A. The consulting physician then conducted a patient interview, performed a physical examination, reviewed the medical records, provided a consultation opinion, and formally executed the consultation within the HIS. Within this process, Departments A and B constituted 1 distinct department consultation pair, and this sequence of actions was recorded as 1 valid consultation (Figure 1).

Per capita consultation request intensity of the department was defined as the ratio of the total annual number of consultation requests made by a department to the total annual number of inpatients admitted to that department.

Among the participating departments, 2 dedicated consultation groups were included: the Internal Medicine Consultation Service and the Surgery Consultation Service. The Internal Medicine Consultation Service comprises physicians specifically dedicated to handling internal medicine consultations. All routine internal medicine consultation requests within the hospital are initially routed to this service for preliminary screening. For routine internal medicine conditions, the Internal Medicine Consultation Service physician directly performs the consultation. For complex conditions necessitating specialized internal medicine expertise, the Internal Medicine Consultation Service physician transfers the consultation request to the relevant internal medicine subspecialty department, which subsequently becomes the receiving department.

The Surgery Consultation Service consists of surgeons dedicatedly responsible for coordinating hospital-wide consultations for biopsies of superficial soft tissue lesions. All other surgical consultation requests are submitted directly to the pertinent surgical subspecialty department.

It should be noted that several participating departments, namely the Internal Medicine Consultation Service, Surgery Consultation Service, Clinical Nutrition Department, Anesthesiology Department, Psychological Medicine Department, Rehabilitation Medicine Department, and Allergy Department, do not operate inpatient wards. The aforementioned departments are classified as Platform Support Departments, which means they do not have dedicated inpatient wards. Their main function is to provide professional technical service support to other clinical departments throughout the hospital, such as imaging diagnosis, anesthesia assessment, nutritional support, and rehabilitation treatment. Consequently, their consultation request volume was inherently zero.

### Conditions and Design

This was a retrospective cohort study using real-world data. This study was conducted at PUMCH, a leading tertiary academic medical center in Beijing, China. As a national referral center for complex and critical illnesses, PUMCH ranks number 1 in the National Tertiary Public Hospital Performance Evaluation. PUMCH has an inpatient bed capacity of more than 2000 beds, with an average occupancy rate of approximately 90.4%. In 2024, the hospital recorded a total of 126,869 inpatient discharges. Additionally, it handles an annual outpatient volume of around 4.27 million visits and an emergency department volume of approximately 287,000 visits. The hospital features a comprehensive department configuration, high patient volumes, and manages diverse and complex clinical cases. Notably, PUMCH ranks first nationally in Case Mix Index, reflecting its role in handling severe and complicated conditions, making it highly representative for studying multidisciplinary collaborative care [43].

### Ethical Considerations

This retrospective study used deidentified data from routine hospital operations. Institutional policy granted ethics exemption (exemption number I-25ZM0094); the approval document is included in the supplementary materials. A waiver of informed consent was approved, as data were collected under standard care protocols allowing deidentified secondary use. All data were anonymized; no personal identifiers were accessible. The study complied with privacy regulations. No compensation was provided. No identifiable images are included; therefore, consent for image publication was not required.

### Analytic Strategy

Consultation-related data were exported from the HIS. Statistical analysis was performed using Microsoft Excel and SPSS (version 27.0; IBM Corp). Continuous variables, such as consultation volumes, were described using medians and IQRs, as the data exhibited a nonnormal distribution based on the Kolmogorov-Smirnov test. Heatmap analysis was performed using Python for data processing. Using the requesting department as row labels and the receiving department as column labels, a pivot table was created to sum the number of consultations, generating an initial consultation matrix. The data underwent log2 transformation for standardization. The Morpheus visualization tool was used to generate the heatmap. Different color gradients (blue-white-red) represent different standardized consultation frequencies: the blue end represents low consultation frequency, white represents the median value, and the red end represents high consultation frequency.

## Results

### Descriptive Statistics of the Consultation Dataset

**Figure 2.**
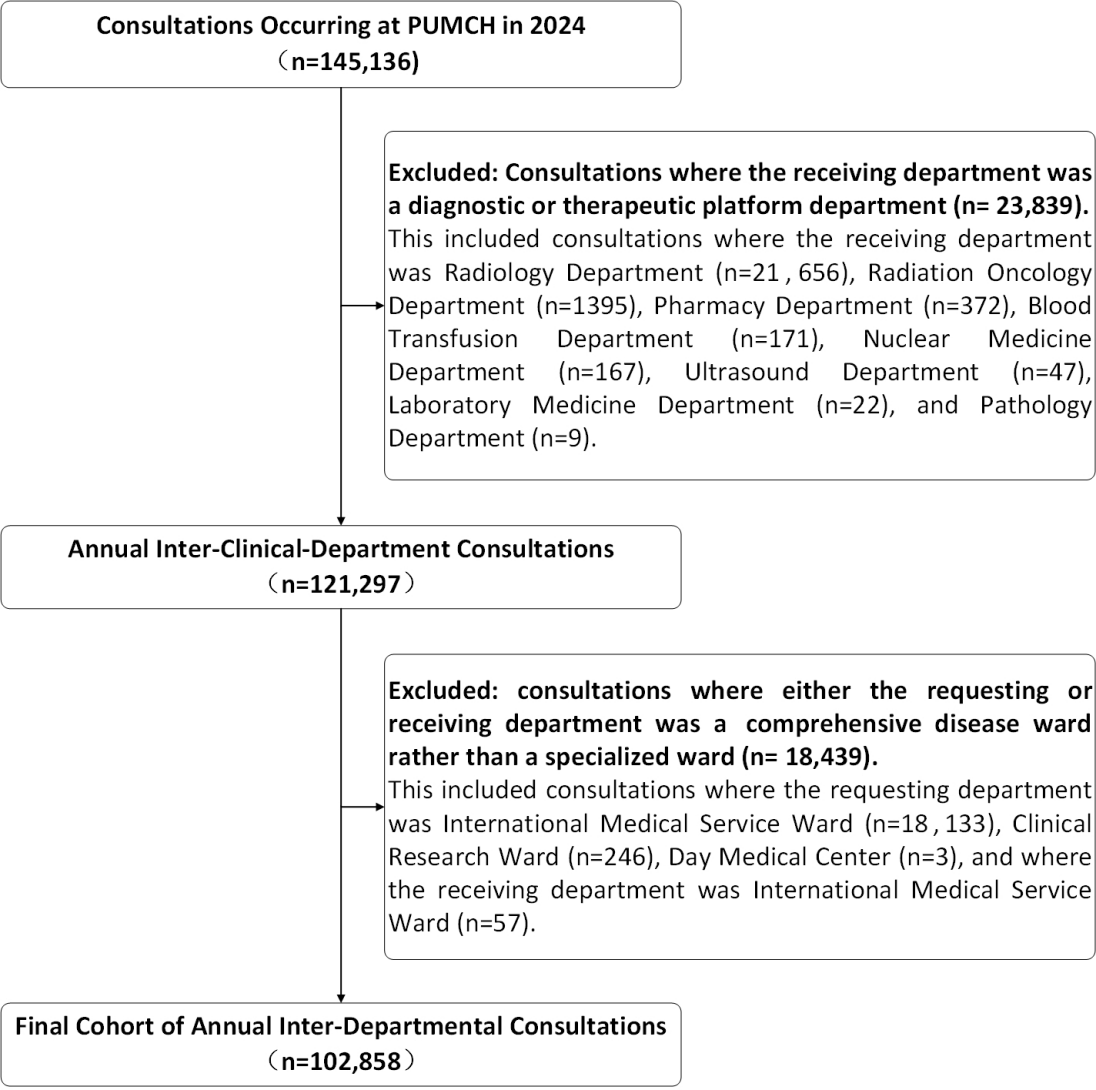
Study flowchart for the inclusion of interdepartmental consultation records at Peking Union Medical College Hospital (PUMCH) (2024). Retrospective study at PUMCH (Beijing, China) from January 1 to December 31, 2024, involving inpatients and emergency patients. After exclusions (diagnostic platform departments and comprehensive disease units), 102,858 valid consultations from 42 clinical departments were included.

The study analyzed 102,858 interdepartmental consultations across 42 clinical departments at PUMCH in 2024 (Figure 2). The descriptive statistics of the consultation dataset are summarized in Table 1.

**Table 1.** Hospital characteristics and consultation dataset overview (retrospective study, Peking Union Medical College Hospital, Beijing, 2024).

Category and variable	Value
Hospital characteristics	
Inpatient bed capacity (beds), n	2182
Average bed occupancy rate, %	90.40
Annual outpatient visits, n	4,273,846
Annual emergency visits, n	287,085
Annual inpatient discharges, n	126,869
Consultation dataset overview	
Total consultations, n	102,858^a^
Inpatient consultations, n (%)	83,160^a^ (80.85)
Emergency consultations, n (%)	19,698^a^ (19.15)
Clinical departments included, n	42^a^
Surgical departments, n (%)	17^a^ (40.47)
Nonsurgical departments, n (%)	25^a^ (59.52)
Department consulting pairs, n	1211^a^
Consultation volume distribution, median (IQR; range)	
Annual consultation requests per department (n=34)	1899.5 (1359.5‐3759.25; 250‐19,698)^a^
Annual consultation receptions per department (n=42)	1886.0 (521.25‐4056.75; 6‐10,428)^a^
Annual consultation volume per department pair (n=1211)	27.0 (7.0‐85.0; 1‐3094)

a Number of departments or consulting pairs.

### Analysis of Consultation Requesting and Receiving by Departments

**Figure 3.**
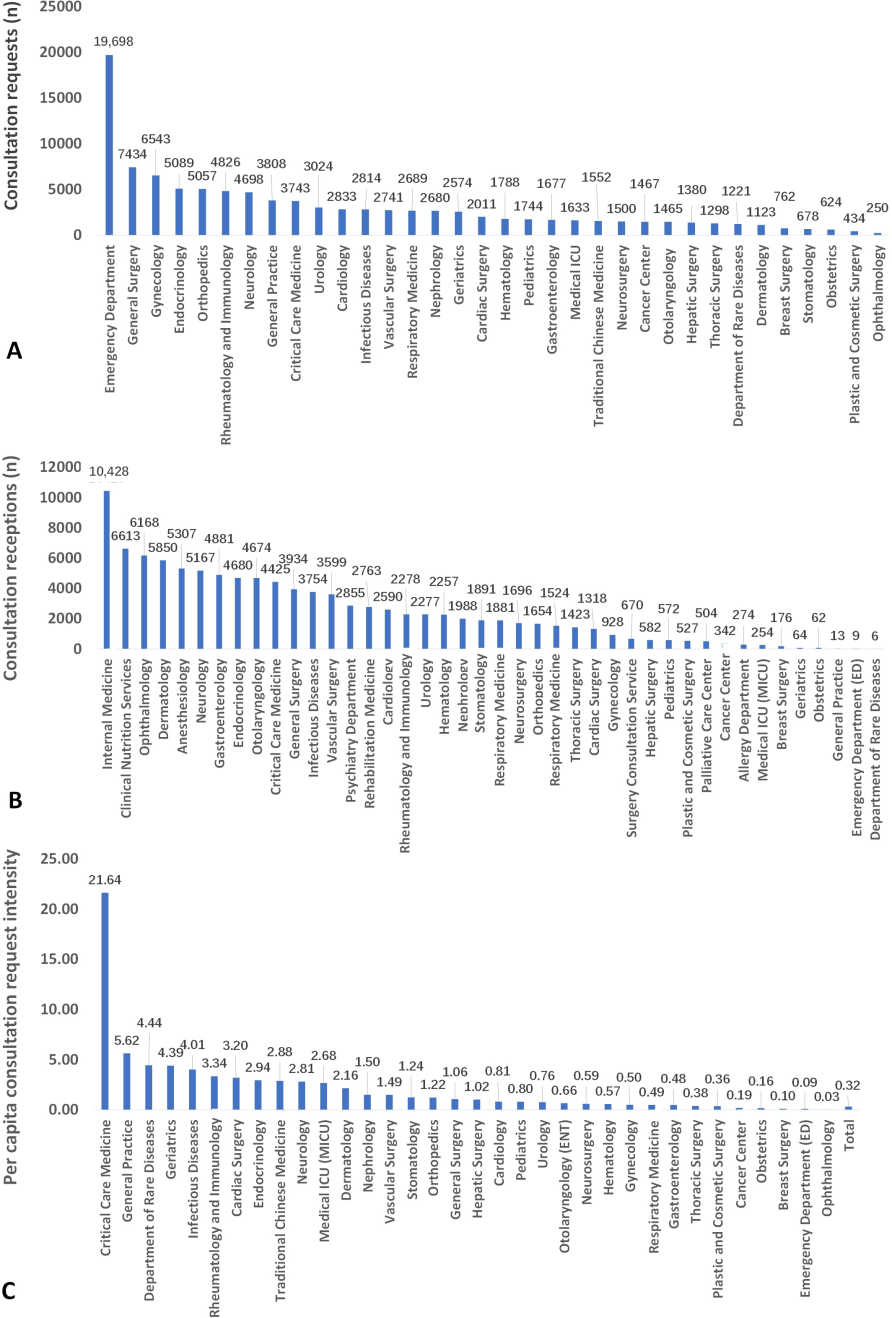
Departmental consultation volume and request intensity Peking Union Medical College Hospital (2024). (A) Number of consultation requests made by each department. (B) Number of consultations received by each department. (C) Per capita request intensity (annual requests/annual inpatient admissions). Data were derived from 102,858 interdepartmental consultations for inpatients and emergency patients. ICU: intensive care unit; TCM: traditional Chinese medicine.

The number of consultation requests and acceptances among the 42 departments at PUMCH in 2024 are detailed in Table 2 and Figure 3.

**Table 2.** Consultation requesting and receiving status by department at Peking Union Medical College Hospital in 2024 (N=102,858 consultations, sorted by total consultation volume: requested + received, descending order).

Department	Category	Consultation requests, n	Consultations received, n	Total consultation volume (requests + received), n (%)	Annual inpatient admissions, n	Per capita consultation request intensity
Emergency Department	Other	19,698	9	19,707 (9.58)	222,666	0.09
General Surgery	Surgery	7434	3934	11,368 (5.53)	6999	1.06
Internal Medicine Consultation Service	Internal Medicine	0	10,428	10,428 (5.07)	0	N/A^a^
Neurology	Internal Medicine	4698	5167	9865 (4.80)	1674	2.81
Endocrinology	Internal Medicine	5089	4680	9769 (4.75)	1729	2.94
Critical Care Medicine	Other	3743	4425	8168 (3.97)	173	21.64
Gynecology	Women & Kids	6543	928	7471 (3.63)	13,027	0.5
Rheumatology and Immunology	Internal Medicine	4826	2278	7104 (3.45)	1443	3.34
Dermatology	Other	1123	5850	6973 (3.39)	521	2.16
Orthopedics	Surgery	5057	1654	6711 (3.26)	4141	1.22
Clinical Nutrition	Other	0	6613	6613 (3.21)	0	N/A
Infectious Diseases	Internal Medicine	2814	3754	6568 (3.19)	701	4.01
Gastroenterology	Internal Medicine	1677	4881	6558 (3.19)	3526	0.48
Ophthalmology	Other	250	6168	6418 (3.12)	7986	0.03
Vascular Surgery	Surgery	2741	3599	6340 (3.08)	1837	1.49
Otolaryngology	Other	1465	4674	6139 (2.98)	2215	0.66
Cardiology	Internal Medicine	2833	2590	5423 (2.64)	3477	0.81
Anesthesiology	Other	0	5307	5307 (2.58)	0	N/A
Urology	Surgery	3024	2277	5301 (2.58)	3962	0.76
Nephrology	Internal Medicine	2680	1988	4668 (2.27)	1784	1.5
Respiratory Medicine	Internal Medicine	2689	1881	4570 (2.22)	5489	0.49
Hematology	Internal Medicine	1788	2257	4045 (1.97)	3138	0.57
General Medicine	Internal Medicine	3808	13	3821 (1.86)	677	5.62
Cardiac Surgery	Surgery	2011	1318	3329 (1.62)	629	3.2
Neurosurgery	Surgery	1500	1696	3196 (1.55)	2538	0.59
Traditional Chinese Medicine	Other	1552	1524	3076 (1.50)	538	2.88
Psychological Medicine	Other	0	2855	2855 (1.39)	0	N/A
Rehabilitation Medicine	Other	0	2763	2763 (1.34)	0	N/A
Thoracic Surgery	Surgery	1298	1423	2721 (1.32)	3383	0.38
Geriatrics	Internal Medicine	2574	64	2638 (1.28)	586	4.39
Stomatology	Other	678	1891	2569 (1.25)	546	1.24
Pediatrics	Women and Kids	1744	572	2316 (1.13)	2173	0.8
Liver Surgery	Surgery	1380	582	1962 (0.95)	1355	1.02
Critical Care Medicine	Other	1633	254	1887 (0.92)	610	2.68
Oncology Medical Center	Internal Medicine	1467	342	1809 (0.88)	7589	0.19
Rare Diseases	Internal Medicine	1221	6	1227 (0.60)	275	4.44
Plastic Surgery	Surgery	434	527	961 (0.47)	1216	0.36
Breast Surgery	Surgery	762	176	938 (0.46)	7552	0.1
Obstetrics	Women and Kids	624	62	686 (0.33)	3944	0.16
Surgery Consultation Service	Surgery	0	670	670 (0.33)	0	N/A
Palliative Care Center	Other	0	504	504 (0.24)	0	N/A
Allergy	Other	0	274	274 (0.13)	0	N/A
Total		102,858	102,858	205,716 (100)	320,099	N/A

a N/A: not applicable.

A total of 34 departments requested consultations, amounting to 102,858 requests. The top 10 departments by number of consultation requests were Emergency Department (n=19,698, 19.15%), General Surgery (n=7434, 7.23%), Gynecology (n=6543, 6.36%), Endocrinology (n=5089, 4.95%), Orthopedics (n=5057, 4.92%), Rheumatology and Immunology (n=4826, 4.69%), Neurology (n=4698, 4.57%), General Medicine (n=3808, 3.70%), and Critical Care Medicine (n=3743, 3.64%). Seven departments (n=7, 20.59%) had annual consultation requests exceeding 4000 consultations, accounting for 53,345 (51.86%) consultations of the hospital’s total consultation volume.

All 42 departments received consultations, totaling 102,858 acceptances. The top 10 departments by number of consultations received were Internal Medicine Consultation Service (n=10,428, 10.14%), Clinical Nutrition (n=6613, 6.43%), Ophthalmology (n=6168, 6.00%), Dermatology (n=5850, 5.69%), Anesthesiology (n=5307, 5.16%), Neurology (n=5167, 5.02%), Gastroenterology (n=4881, 4.75%), Endocrinology (n=4680, 4.55%), Otolaryngology (n=4674, 4.54%), and Critical Care Medicine (n=4425, 4.30%). Ten (23.81%) departments received over 4000 consultations annually, accounting for 58,193 (56.58%) consultations of the hospital’s total consultation volume.

The consultation volume received by the internal medicine system was significantly higher than that by the surgical system (40,328 vs 17,856 consultations). Within the internal medicine system, apart from the Internal Medicine Consultation Service (n=10,428, 25.85% of internal medicine consultations), the top 3 departments by volume received were Gastroenterology (n=4881, 12.10%), Endocrinology (n=4680, 11.60%), and Infectious Diseases (n=3754, 9.31%).

The top 10 departments by highest per capita consultation request intensity were Critical Care Medicine (21.64), General Medicine (5.62), Rare Diseases (4.44), Geriatrics (4.39), Infectious Diseases (4.01), Rheumatology and Immunology (3.34), Cardiac Surgery (3.20), Endocrinology (2.94), Traditional Chinese Medicine (2.88), and Neurology (2.81). The hospital-wide average per capita consultation request intensity (total annual consultation requests / total inpatient admissions) was 0.32.

### Analysis of Interdepartmental Consultation Associations

Consultation requests and acceptances formed departmental consultation pairs. In 2024, PUMCH had 1211 unique consultation pairs. Details of pairs with an annual consultation volume of ≥500 consultations are shown in Table 3. Consultation pairs with ≥700 consultations numbered 18 (1.49%) pairs, accounting for 22,302 (21.68%) consultations (Multimedia Appendix 1); pairs with ≥500 consultations numbered 31 (2.56%) pairs, accounting for 30,276 (29.43%) consultations; pairs with ≥300 consultations numbered 65 (5.37%) pairs, accounting for 43,221 (42.02%) consultations.

**Table 3.** High-frequency department consultation pairs (annual volume ≥500)^a^.

Requesting department	Receiving department	Main consultation content	Consultations, n (%)
Emergency Department	Internal Med Consult^b^	Internal disease management	3094 (3.01)
Emergency Department	Dermatology	Rashes, skin lesions	2840 (2.76)
Emergency Department	Gastroenterology	Acute abdomen, gastrointestinal bleeding	1753 (1.70)
Emergency Department	General Surgery	Acute abdomen, abdominal masses	1451 (1.41)
Emergency Department	Neurology	Cerebrovascular events, dizziness, headache	1386 (1.35)
Endocrinology	Ophthalmology	Diabetic retinopathy, thyroid eye disease	1287 (1.25)
General Surgery	Otolaryngology	Preoperative thyroid airway assessment	1225 (1.19)
General Surgery	Clinical Nutrition	Perioperative nutrition support	1032 (1)
Gynecology	Critical Care Medicine	Postoperative management	983 (0.96)
Orthopedics	Internal Med Consult	Perioperative internal disease management	955 (0.93)
General Surgery	Internal Med Consult	Perioperative internal disease management	940 (0.91)
Emergency Department	Vascular Surgery	Acute arterial embolism, venous thrombosis	928 (0.90)
General Surgery	Gastroenterology	Collaborative treatment of digestive diseases	790 (0.77)
Gynecology	Internal Med Consult	Perioperative internal disease management	778 (0.76)
General Surgery	Critical Care Medicine	Postoperative management and critical care	722 (0.70)
Emergency Department	Neurosurgery	Acute cerebrovascular events	721 (0.70)
Gynecology	Anesthesiology	Preoperative anesthesia assessment	714 (0.69)
Endocrinology	Clinical Nutrition	Metabolic disease nutrition management	703 (0.68)
Orthopedics	Rehabilitation Medicine	Postoperative rehabilitation management	686 (0.67)
Oncology Medical Center	Clinical Nutrition	Nutritional support for patients with cancer	681 (0.66)
Rheumatology Immunology	Ophthalmology	Immune-related eye disease	678 (0.66)
Neurology	Internal Med Consult	Comorbid internal disease management	662 (0.64)
Urology	Internal Med Consult	Comorbid internal disease management	657 (0.64)
General Surgery	Anesthesiology	Preoperative anesthesia assessment	640 (0.62)
Gynecology	General Surgery	Collaborative surgery	635 (0.62)
Cardiac Surgery	Critical Care Medicine	Post-cardiac surgery management	622 (0.60)
Pediatrics	Ophthalmology	Pediatric eye disease	576 (0.56)
Emergency Department	Endocrinology	Metabolic disease management	567 (0.55)
Emergency Department	Otolaryngology	Ear, nose, and throat emergencies	560 (0.54)
Vascular Surgery	Internal Med Consult	Comorbid internal disease management	506 (0.49)
Urology	Anesthesiology	Preoperative anesthesia assessment	504 (0.49)

a Based on 102,858 interdepartmental consultations at Peking Union Medical College Hospital in 2024, showing main content, count, and percentage of each requesting-receiving department pair.

b Internal med consult: internal medicine consultation service.

The Emergency Department was the primary requesting department. Its highest-volume consultation pairs were with the Internal Medicine Consultation Service (3094/102,858, 3.01%), Dermatology (2840/102,858, 2.76%), Gastroenterology (1753/102,858, 1.70%), General Surgery (1451/102,858, 1.41%), and Neurology (1386/102,858, 1.35%), primarily involving management of diseases in emergency patients (Table 3). As the largest consultation-receiving department, the Internal Medicine Consultation Service had relatively balanced demand from various surgical departments. Among departments with platform support functions, Anesthesiology had relatively balanced demand from surgical departments, whereas Clinical Nutrition and Rehabilitation Medicine showed strong specific associations. For example, the main sources of demand for Clinical Nutrition were General Surgery (1032/6613, 15.61% of its received consultations), Endocrinology (703/6613, 10.63%), and Oncology Medical Center (681/6613, 10.30%), primarily for perioperative nutrition management in patients undergoing gastrointestinal surgery and nutritional support and guidance for patients with metabolic diseases and cancer. The main sources of demand for Rehabilitation Medicine were Orthopedics (686/2763, 24.83%), Neurology (396/2763, 14.33%), and Geriatrics (215/2763, 7.78%), primarily for postoperative rehabilitation guidance for orthopedic patients, patients with neurological damage, older adults, and patients with sarcopenia.

Other notable disease-specific high-volume pairs included Endocrinology-Ophthalmology, primarily for diabetic eye disease and thyroid eye disease (1287/102,858, 1.25%); General Surgery-Otolaryngology, mainly for preoperative thyroid airway assessment (n=1225, 1.19%); General Surgery-Clinical Nutrition, primarily for perioperative support (n=1032, 1.00%); Endocrinology-Clinical Nutrition, mainly for metabolic disease management (n=703, 0.68%); Orthopedics-Rehabilitation Medicine, primarily for postoperative rehabilitation (n=686, 0.67%); Oncology Medical Center-Clinical Nutrition, mainly for nutrition support in patients with cancer (n=681, 0.66%); and Rheumatology Immunology-Ophthalmology, primarily for immune-related eye disease (n=678, 0.66%; Table 3). Recurring clinical scenarios generated these stable, predictable consultation pathways.

**Figure 4.**
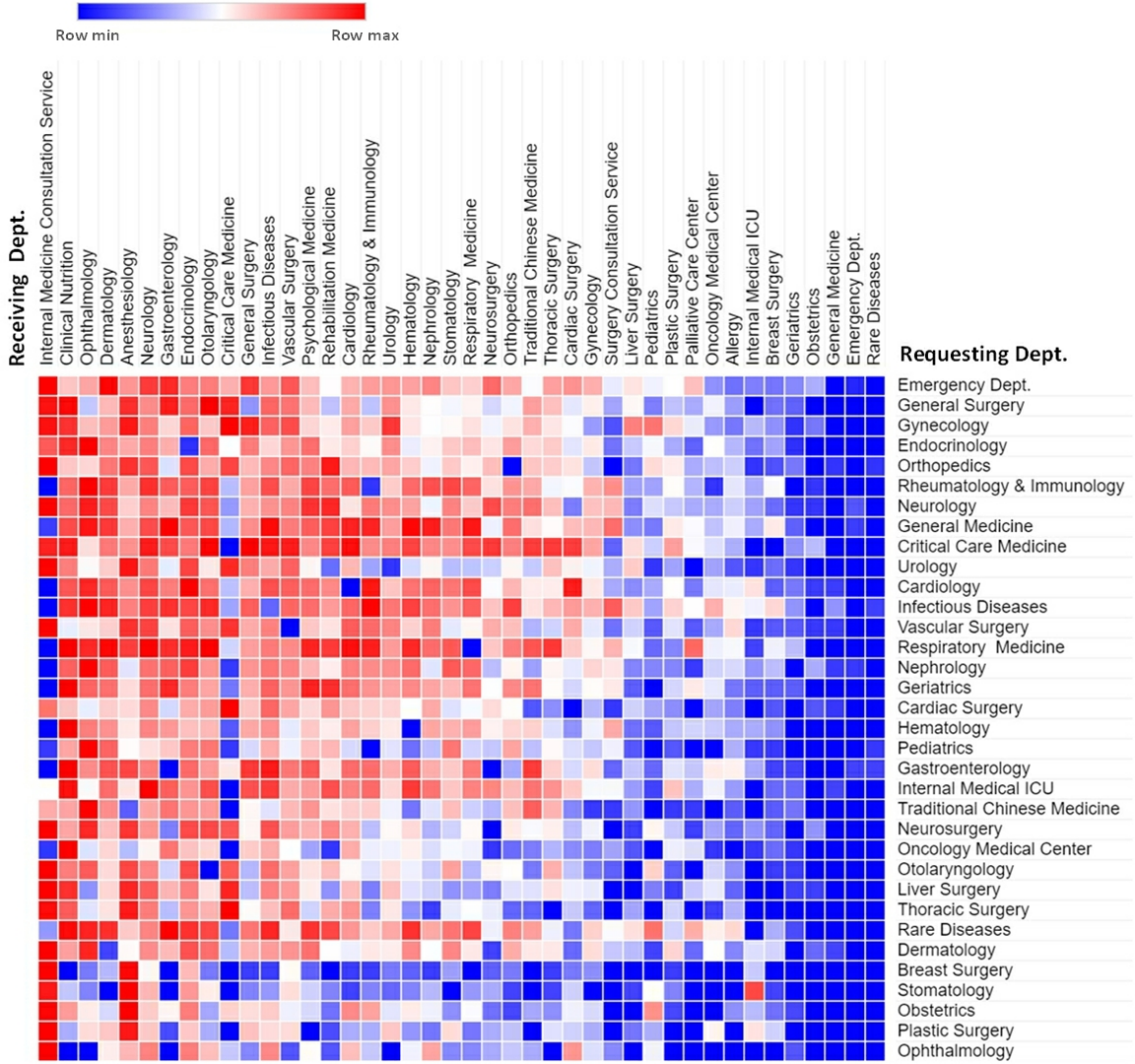
Heatmap analysis of interdepartmental consultation associations. Retrospective study at Peking Union Medical College Hospital (Beijing, 2024) of 102,858 consultations. Rows=requesting departments (sorted by total requests descending); columns=receiving departments (sorted by total receptions descending). Color gradient (blue-white-red) indicates standardized consultation frequency (blue=low, red=high). Dept.: department; ICU: intensive care unit.

Heatmap analysis showed the consultation associations between departments in 2024 (Figure 4). Overall, it shows significant interdepartmental differences, reflecting the collaborative characteristics of different departments during medical service delivery. Color distribution: The Blue-White-Red gradient was used, with red indicating high-frequency consultations (maximum: Emergency Department → Internal Medicine Consultation Service, 3094 consultations), blue indicating low-frequency consultations (minimum: 1‐5 consultations between multiple departments), and white representing the transition zone corresponding to medium consultation volumes.

The most prominent red areas were concentrated at the intersections of the Emergency Department and Internal Medicine Consultation Service (n=3094), Emergency Department and Dermatology (n=2840), and Emergency Department and Gastroenterology (n=1753). Row pattern analysis (requesting departments): the Emergency Department row shows an overall reddish hue, indicating its high-frequency consultations with multiple departments. Rows for departments like Breast Surgery and Plastic Surgery were predominantly blue, indicating generally low consultation participation frequency. Column pattern analysis (receiving departments): the Internal Medicine Consultation Service column shows a distinct red stripe, indicating high consultation volumes received from the Emergency Department (n=3094), General Surgery (n=940), Orthopedics (n=955), etc. Columns for Clinical Nutrition and Anesthesiology showed mixed colors, indicating significant variation in consultation demand from different departments.

Furthermore, the heatmap allows intuitive visualization of interactions with target departments. For example, when Critical Care Medicine was the receiving department, its interactions with Cardiac Surgery (n=622), Gynecology (n=983), and General Surgery (n=722) appeared orange to red, indicating frequent consultations. When Ophthalmology was the receiving department, its interactions with Endocrinology (n=1287), Pediatrics (n=576), and Nephrology (n=488) were prominent.

## Discussion

### Principal Findings

In this real-world analysis of interdepartmental consultations, we systematically mapped the collaboration network of multiple clinical departments from a hospital-wide perspective for the first time. Unlike previous studies limited to single departments or specific diseases, this study reveals three core findings that support our original hypotheses. First, consultation demand is highly concentrated: the Emergency Department requests the most consultations, and the Internal Medicine Consultation Service receives the most. Second, collaboration follows a Pareto-like pattern, where a small number of department pairs account for a large share of all consultations. Third, high volume pairs are consistently disease driven, such as Endocrinology-Ophthalmology for diabetic and thyroid eye diseases, General Surgery-Otolaryngology for preoperative thyroid airway assessment, and Orthopedics-Rehabilitation Medicine for postoperative rehabilitation. These stable collaboration pathways indicate that interdepartmental consultations are not random but cluster around specific clinical needs [10,33,44-46].

### Similarity of Results

The high number of consultation requests from the Emergency Department is consistent with previous studies highlighting its role as a gateway for complex, undifferentiated patients who often need rapid specialist input [12,14,27]. Notably, the Emergency Department’s consultation needs were broad, involving internal medicine, surgery, and multiple specialties. Its main consultation flows included the Internal Medicine Consultation Service (comorbidities), Dermatology (rashes, soft tissue infections), and Gastroenterology (acute abdomen, gastrointestinal bleeding)—all common emergency scenarios requiring specialist input.

The high-frequency pairs identified in this study align well with successful multidisciplinary team applications reported in the literature [7,31-33,45,47]. For example, the Endocrinology-Ophthalmology pair for diabetic retinopathy and thyroid eye disease is consistent with national and international multidisciplinary team (MDT) guidelines recommending joint screening and management [45]. In the perioperative setting for thyroid surgery, the General Surgery-Otolaryngology pair for preoperative laryngeal assessment represents a critical safety standardization. The multiple high-frequency pairs involving Clinical Nutrition (with General Surgery, Oncology Medical Center, and Endocrinology) address perioperative nutritional support, cancer nutrition management, and metabolic disease nutrition intervention, respectively—all consistent with current MDT practices in comprehensive cancer care and metabolic disease management [33,44,46]. For orthopedic surgery, the Orthopedics-Rehabilitation Medicine pair for postoperative rehabilitation embodies the integrated “surgery-rehabilitation-functional recovery” model.

### Interpretation

The above findings have several practical implications. First, the high concentration of consultation demand suggests that resources should be focused on “top-tier” needs. The Emergency Department, as the largest requester, could benefit from dedicated on-site specialist consultation positions or, for scenarios that do not require bedside visits (eg, dermatology), telemedicine consultations to reduce response times. Previous studies have shown that delays in emergency consultations are a key factor affecting length of stay and outcomes [17,25,27,28,48-52].

Second, the disease specificity of high-frequency pairs provides a direct basis for designing standardized MDT pathways [32,33,53]. Traditional MDT models rely on fixed-time, fixed-location meetings, which are resource-intensive and difficult to scale. This study found that consultation volume is highly concentrated in a limited set of recurring clinical scenarios. Hospitals can therefore develop “lightweight” MDT pathways for these high-frequency scenarios, such as endocrinology-ophthalmology joint clinics, orthopedics-rehabilitation joint rounds, or preoperative combined assessment pathways, shifting MDT from a “specialty-only” to a “routine” model [45,53,54].

Third, the prominent role of platform departments (Clinical Nutrition, Anesthesiology, Rehabilitation Medicine, etc) as major consultation recipients reflects the ongoing trend toward integrated care. This suggests that hospital planning and resource allocation should give due attention to the capacity building and spatial placement of platform departments—for example, locating Clinical Nutrition and Rehabilitation Medicine adjacent to surgical wards, placing preoperative anesthesia assessment clinics upstream of surgical outpatient areas, or establishing admission preparation centers—to reduce unnecessary intrahospital movement and consultation waiting times [18,44,46,54,55].

Fourth, the large number of consultations directed to the Internal Medicine Consultation Service also deserves a nuanced interpretation. It may act as an efficient gatekeeper, streamlining access to subspecialties [8,13,30,56-58]; it could also reveal knowledge gaps in requesting departments, pointing to opportunities for targeted education [42,54]; or it might represent a vital integrative function in an era of deep subspecialization, arguing for formally recognizing such roles in coordinated care models [30,38,49]. Delving into the specific context of such high-volume interactions can thus inform more targeted strategies to enhance both the efficiency and effectiveness of interdepartmental consultation.

### Generalizability

The analytical framework and findings of this study have broad generalizability. Although the data originated from a single large tertiary hospital, the revealed “Pareto-like + disease-driven” collaboration pattern is theoretically applicable to various health care institutions. Any general hospital with a reasonable specialty mix can extract consultation records from its HIS, construct a similar departmental collaboration network, identify its own high-frequency pairs, and guide resource optimization accordingly.

Several high-frequency pairs identified in this study, such as Endocrinology-Ophthalmology, Orthopedics-Rehabilitation Medicine, and Clinical Nutrition with surgical departments, are likely to be cross-institutionally generalizable because these collaborations are driven by the inherent logic of disease management rather than by institution-specific arrangements. Hospitals of different levels and regions can therefore use our findings as a reference to prioritize establishing standardized collaborative pathways for these “universal” high-frequency scenarios.

More importantly, the analytical approach described here can directly inform 2 types of applications. First, in workflow optimization within smart hospital initiatives—for example, establishing MDT joint clinics for high-frequency consultation pairs, or prioritizing e-consultations for scenarios that do not require bedside visits. Second, in the spatial layout planning of new hospitals—by preidentifying high-frequency collaboration pairs, closely collaborating departments can be colocated to shorten physical consultation pathways [7,38,41]. It is worth noting that this study was initially motivated by the planning of a new medical center focused on complex and rare diseases, where consultation patterns were considered as one of the factors influencing spatial layout and workflow.

### Limitations

This study has several limitations. As a single-center retrospective analysis based on a large tertiary hospital, its consultation patterns are influenced by the hospital’s specific disciplinary structure, case mix, and internal processes. Therefore, the generalizability of the conclusions requires validation against the specific contexts of other institutions. Additionally, the current analysis primarily focused on consultation volume and did not encompass multi-dimensional indicators such as consultation duration, the quality of consultation opinions, or patient outcomes. Future research that links consultation network patterns to clinical and operational outcomes would be valuable.

### Conclusions

In summary, this study goes beyond traditional single-disease or single-department analyses to provide a systematic, hospital-wide picture of interdepartmental collaboration. The demonstration that consultations are highly concentrated, follow a Pareto-like distribution, and cluster around disease-specific pairs offers a data-driven foundation for rethinking consultation processes. For hospital administrators and clinicians, the practical implications of this study are threefold. First, prioritize resources: allocate limited personnel, space, and information technology resources toward high-volume collaborative pairs. Second, design standardized MDT pathways: establish rapid MDT channels or joint clinics for high-frequency scenarios such as diabetic eye disease, preoperative thyroid assessment, and postoperative rehabilitation. Third, implement spatial and digital interventions: consider spatial proximity for high-frequency collaborating pairs in new hospital planning and promote e-consultations for appropriate scenarios. More broadly, the analytical framework we present can serve as a model for other institutions to use their own real-world consultation data to improve care coordination, boost operational efficiency, and ultimately enhance patient flow and outcomes.

## Supplementary material

10.2196/81670 Multimedia Appendix 1 Departmental consultation pairs with annual consultation volume ≥700 (Peking Union Medical College Hospital, 2024).

## Abbreviations

HIS: Hospital Information System
MDT: multidisciplinary team
PUMCH: Peking Union Medical College Hospital

## References

[1] Saint-Pierre C, Herskovic V, Sepúlveda M (2018). Multidisciplinary collaboration in primary care: a systematic review. Fam Pract.

[2] Sharshar T, Grimaldi-Bensouda L, Siami S (2024). A randomized clinical trial to evaluate the effect of post-intensive care multidisciplinary consultations on mortality and the quality of life at 1 year. Intensive Care Med.

[3] Wang Z, Lai L, Zhang X, Sun Y (2025). Determinants of severely prolonged emergency department stays among older adults: a retrospective comparative analysis. J Nurs Manag.

[4] Marijon E, Narayanan K, Smith K (2023). The Lancet Commission to reduce the global burden of sudden cardiac death: a call for multidisciplinary action. Lancet.

[5] de Gans S, Penturij-Kloks M, Scheele F, van de Pol M, van der Zwaard B, Keijsers C (2023). Combined *inter*professional and *intra* professional clinical collaboration reduces length of stay and consultations: a retrospective cohort study on an intensive collaboration ward (ICW). J Interprof Care.

[6] Blumenthaler AN, Bruera E, Badgwell BD (2023). Palliative and supportive care consultation for patients with malignant gastrointestinal obstruction is associated with broad interdisciplinary management. Ann Surg.

[7] Engels RC, Harrop CM, Ackermann LL (2024). Medical consultation and comanagement. Med Clin North Am.

[8] Hou M, Gong X, Chang W (2021). Will multidisciplinary collaboration reduce the disability rate of diabetic foot (2009-2019)?-A study based on the perspective of organizational reform. Front Public Health.

[9] Pillay B, Wootten AC, Crowe H (2016). The impact of multidisciplinary team meetings on patient assessment, management and outcomes in oncology settings: a systematic review of the literature. Cancer Treat Rev.

[10] Raat W, Smeets M, Vandewal I (2021). Cardiologists’ perceptions on multidisciplinary collaboration in heart failure care - a qualitative study. BMC Health Serv Res.

[11] Meyer-Schwickerath C, Weber C, Hornuss D (2024). Complexity of patients with or without infectious disease consultation in tertiary-care hospitals in Germany. Infection.

[12] Brick C, Lowes J, Lovstrom L (2014). The impact of consultation on length of stay in tertiary care emergency departments. Emerg Med J.

[13] Dadeh A, Phunyanantakorn P (2020). Factors affecting length of stay in the emergency department in patients who presented with abdominal pain. Emerg Med Int.

[14] Sun BC, Hsia RY, Weiss RE (2013). Effect of emergency department crowding on outcomes of admitted patients. Ann Emerg Med.

[15] Uddin S, Hossain L, Kelaher M (2012). Effect of physician collaboration network on hospitalization cost and readmission rate. Eur J Public Health.

[16] Ataman MG, Sariyer G, Saglam C, Karagoz A, Unluer EE (2023). Factors relating to decision delay in the emergency department: effects of diagnostic tests and consultations. Open Access Emerg Med.

[17] Lorente-Lavirgen AI, Bernabeu-Wittel J, Pulpillo-Ruiz Á, de la Torre-García JM, Conejo-Mir J (2013). Inpatient dermatology consultation in a Spanish tertiary care hospital: a prospective cohort study [Article in English, Spanish]. Actas Dermosifiliogr.

[18] Ruiz de Assín Valverde A, Alfaro Martínez JJ, López García MC (2024). Evolution of interconsultal activity to endocrinology and nutrition in hospitalization floor in a third level hospital. Endocrinol Diabetes Nutr (Engl Ed).

[19] Yazici DA, Satici C, Bahadir A (2025). AI-based assessment of pulmonology inpatient consultation note completeness: predicting documentation gaps and response delays. Int J Med Inform.

[20] Ma H, Li H, Xu T (2024). Multidisciplinary team quality improves the survival outcomes of locally advanced rectal cancer patients: a post hoc analysis of the STELLAR trial. Radiother Oncol.

[21] Egleston BL, Bleicher RJ, Fang CY, Galloway TJ, Vucetic S (2023). Benefits versus drawbacks of delaying surgery due to additional consultations in older patients with breast cancer. Cancer Rep (Hoboken).

[22] Nadler AD, Eid SM, Kisuule F (2026). Categorizing care delays and their impact on hospital length of stay. Qual Manag Health Care.

[23] Şimşek EK, Kafa B, Haberal B (2025). Preoperative consultations and their effect on surgical delays and mortality in hip fracture surgery. Orthop Surg.

[24] Campbell JM, Yost S, Gautam D (2024). Delays in the diagnosis and surgical treatment of drug-resistant epilepsy: a cohort study. Epilepsia.

[25] Rey-Aldana D, Mazón-Ramos P, Portela-Romero M (2022). Longer-term results of a universal electronic consultation program at the cardiology department of a Galician Healthcare Area. Circ Cardiovasc Qual Outcomes.

[26] Serling-Boyd N, Miloslavsky EM (2020). Enhancing the inpatient consultation learning environment to optimize teaching and learning. Rheum Dis Clin North Am.

[27] Voaklander B, Gaudet LA, Kirkland SW, Keto-Lambert D, Villa-Roel C, Rowe BH (2022). Interventions to improve consultations in the emergency department: a systematic review. Acad Emerg Med.

[28] Yoon SS, Wong DH, Wormwood JB, Reisman JI, Vimalananda VG (2021). Impact of electronic consultation on timeliness and guideline concordance of workups leading to thyroid nodule fine-needle aspiration biopsy. Endocr Pract.

[29] Al-Na’seh MH, Elheet AM, Alhayek AM, Sabri AT, Al Owaidat AK (2024). Optimizing emergency department length of stay and quality of care: a quality improvement project. Cureus.

[30] Montero Ruiz E, Rebollar Merino A, García Sánchez M, Culebras López A, Barbero Allende JM, López Álvarez J (2014). Analysis of in-hospital consultations with the department of internal medicine [Article in English, Spanish]. Rev Clin Esp (Barc).

[31] Rosenblum RE, Ormond E, Smith CW (2023). Institution of standardized consultation criteria to increase early palliative care utilization in older patients with acute leukemia. JCO Oncol Pract.

[32] Arbaugh C, Kimura C, Kin C (2024). Gastrointestinal surgical patient and multidisciplinary healthcare provider beliefs and practices around perioperative nutrition: a mixed-methods study. J Surg Res.

[33] Chen J, Luo AL, Yang L, Wang W, Zhou X, Yang M (2023). Nutrition management by a multidisciplinary team for prevention of nutritional deficits and morbidity following esophagectomy. Braz J Med Biol Res.

[34] Abdel-Hafez A, Jones M, Ebrahimabadi M (2023). Artificial intelligence in medical referrals triage based on clinical prioritization criteria. Front Digit Health.

[35] Job J, Nicholson C, Donald M, Jackson C, Byrnes J (2023). An eConsultant versus a hospital-based outpatient consultation for general (internal) medicine: a costing analysis. BMC Health Serv Res.

[36] Keely E, Guglani S, Mitchell E, Sethuram C, Afkham A, Liddy C (2025). Specialists accessing specialty advice: evaluating utilization, benefits, and impact of care of an e-consultation service. J Telemed Telecare.

[37] Zafar JE, Chan KT, Ryder LJ (2023). Information technology-enhanced telehealth consultations reduce preoperative evaluation center visits in a bariatric surgery population. Healthcare (Basel).

[38] Canetta C, Accordino S, La Boria E (2024). Effects of a medical admission unit on in-hospital patient flow and clinical outcomes. Eur J Intern Med.

[39] Howell E, Bessman E, Kravet S, Kolodner K, Marshall R, Wright S (2008). Active bed management by hospitalists and emergency department throughput. Ann Intern Med.

[40] Pati D, Harvey Jr TE, Redden P, Summers B, Pati S (2015). An empirical examination of the impacts of decentralized nursing unit design. HERD.

[41] Dos Santos VC, Siqueira RM, Godinho-Filho M (2024). Enhancing healthcare operations: a systematic literature review on approaches for hospital facility layout planning. J Health Organ Manag.

[42] Merker L, Conroy S, El-Wakeel H, Laurence N (2023). Streamlining the multi-disciplinary team meeting: the introduction of robust pre-preparation methods and its effect on the length of case discussions. J Multidiscip Healthc.

[43] National Health Commission of the People’s Republic of China (2022). National report on medical service and quality safety 2022 [report in Chinese].

[44] Hartwell JL, Evans DC, Martin MJ (2024). Nutritional support for the trauma and emergency general surgery patient: what you need to know. J Trauma Acute Care Surg.

[45] Lytvyn Y, Felfeli T, Dubrofsky L (2024). Diabetic retinopathy screening integrated in a multidisciplinary diabetes care clinic: a pilot project. Can J Ophthalmol.

[46] Martinuzzi A, Crivelli A, Lopez A (2024). Nutritional support team intervention in surgical ICUs and its effect on nutrition delivery and quality in critically ill patients. Nutrition.

[47] Braam A, van Wijngaarden J, Hilders C, Buljac-Samardzic M (2024). Multidisciplinary collaboration in hospitals via patient- and process-oriented units: a longitudinal study. J Multidiscip Healthc.

[48] Goldberg-Stein S, Varghese J, Wang JJ (2024). Global health initiatives: international physician-to-physician consultation programs. J Am Coll Radiol.

[49] Liu X, Ben Liu Q (2024). Superior medical resources or geographic proximity? The joint effects of regional medical resource disparity, geographic distance, and cultural differences on online medical consultation. Soc Sci Med.

[50] Swanson MB, Miller AC, Ward MM, Ullrich F, Merchant KA, Mohr NM (2021). Emergency department telemedicine consults decrease time to interpret computed tomography of the head in a multi-network cohort. J Telemed Telecare.

[51] Aledia AS, Dangodara AA, Amin AA, Amin AN (2024). Implementation of inpatient electronic consultations during the COVID-19 crisis and its sustainability beyond the pandemic: quality improvement study. J Med Internet Res.

[52] Zheng L, Guggina LM, Zhou XA (2023). Perceptions of telehealth among inpatient consultative dermatology providers and practice patterns during COVID-19. Arch Dermatol Res.

[53] Owens WR, Schmidt JL, Hollier PC, Cole SH, Buchanan EP, Ching JA (2025). Introduction to multidisciplinary clinics and their value. Semin Plast Surg.

[54] Patel M, Gardner TA, White C, Keniston A, Maassen B, Gottenborg E (2023). Interventions to reduce inappropriate physical therapy consultation in the inpatient setting: a quality improvement initiative. J Healthc Qual.

[55] Song IA, Lee K, Lee S, Kim K, Oh TK (2024). Implementation of a multidisciplinary nutritional support team and clinical outcomes in critically ill patients with COVID-19. Clin Nutr.

[56] Liu YC, Schmidt RO, Kapadia NS, Phillips JD, Moen EL (2024). Disparities in access to multidisciplinary cancer consultations and treatment for patients with early-stage non-small cell lung cancer: a SEER-Medicare analysis. Int J Radiat Oncol Biol Phys.

[57] Kawamura R, Harada Y, Yokose M, Hanai S, Suzuki Y, Shimizu T (2023). Survey of inpatient consultations with general internal medicine physicians in a tertiary hospital: a retrospective observational study. Int J Gen Med.

[58] Rosa PRM, Spagnól MF, Rothlisberger L, Gelain MAS, de Brida MS, Teixeira C (1992). Internal medicine consultation for high-risk surgical patients: reflection on hospital mortality and readmission rates in a low-income country. Rev Assoc Med Bras.

