# Supplementary material for: Concentration and Specialty Pair Patterns of Interdepartmental Consultations in Hospitalized Patients Using Real-World Data: Retrospective Cohort Study
Source: J Med Internet Res. 2026 Jul 14;28:e81670. doi: 10.2196/81670 (PMC13367226; doi:10.2196/81670)

Figure 1. Departmental consultation pairs with annual consultation volume ≥700 (Peking Union Medical College Hospital, 2024). Inner ring: requesting departments; outer ring: receiving departments. The 18 pairs shown (≥700 consultations each) accounted for 21.68% (n=22,302) of the total 102,858 consultations in this retrospective study of inpatients and emergency patients. Internal med consult: internal medicine consultation service.


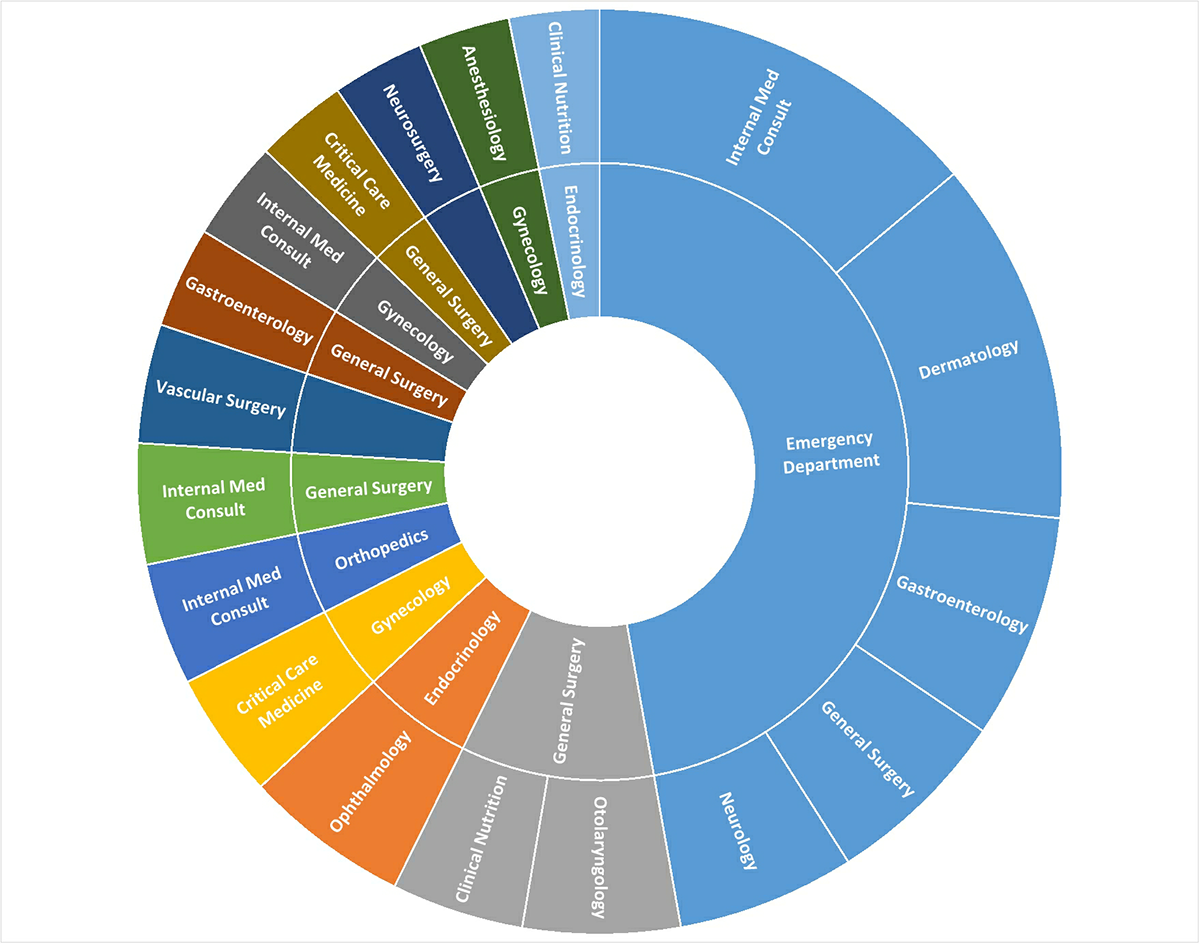

Supplement: Multimedia Appendix 1 [file jmir-v28-e81670-s001.docx]
